# Editorial: Computational mechanism of genetic/evolutionary operator and optimizations in genomic data applications

**DOI:** 10.3389/fgene.2023.1343199

**Published:** 2023-11-29

**Authors:** Jianing Xi, Zhenhua Yu, Wen Shi

**Affiliations:** ^1^ School of Biomedical Engineering, Guangzhou Medical University, Guangzhou, China; ^2^ School of Information Engineering, Ningxia University, Yinchuan, China

**Keywords:** genetics, evolution, optimization, genomics, computational mechanism

## Introduction

The exponential growth of genomic data, driven by advancements in high-throughput sequencing technologies, has precipitated the need for innovative data management solutions (Vandereyken et al., 2023). The challenge extends beyond mere storage to encompass the swift transmission and processing of large datasets, which are essential for timely and effective data analysis and interpretation (Caudai et al., 2021; Yan et al., 2022). The principles of genetics and evolutionary theory have fundamentally explained the emergence and progression of the biological realm, bringing transformative concepts of growth and variation to biology (Moczek et al., 2015). This evolutionary perspective has not only accelerated advancements in genetic research but also significantly propelled other scientific fields forward (Shi et al., 2021; Diaz-Flores et al., 2022). Motivated by genetic and evolutionary theories, researchers have developed numerous computational strategies rooted in genetic and evolutionary operations and stochastic search techniques (Ünal and Başçiftçi, 2022).

In recent times, the application of genomic data has encountered optimization challenges where conventional mathematical approaches fall short (Zhou et al., 2019). Genetic and evolutionary algorithms stand apart from traditional calculus-based and exhaustive methods due to their ability to achieve global optimization with remarkable robustness and broad applicability (Viriyasitavat et al., 2021; Shi et al., 2022). Characterized by self-organization, self-adaptation, and self-learning, these algorithms can adeptly handle complex optimizations irrespective of the problem’s nature (Gheibi et al., 2021; Liu et al., 2022). Genomic data analysis often deals with intricate patterns and complex regulations unsuitable for traditional optimization methods (Hassija et al., 2023; Xi et al., 2023). Integrating genetic and evolutionary algorithm-based complex optimizations in genomic analysis can alleviate bottlenecks in bioinformatics tasks (Xi et al., 2020a; Mandal et al., 2023). Therefore, this research theme focuses on exploring complex optimization challenges in genomic data applications using genetic and evolutionary algorithms (Jiao et al., 2023).

Advancements in genomic sequencing technology have led to an explosion of data, marking the advent of the genomics big data era (Xi et al., 2020b). This surge brings both possibilities and challenges, especially in terms of data storage, processing, and interpretation (Ahmed et al., 2022). The articles in this special edition, titled “Computational Mechanism of Genetic/Evolutionary Operator and Optimizations in Genomic Data Applications,” collectively tackle these issues through cutting-edge computational methods. They delve into the complexities of genomic data and introduce innovative ways to optimize its application across various biological and clinical settings.

## Optimizing genomic data storage and processing

In this Research Topic, the paper titled “Enhancing Genomic Mutation Data Storage Optimization based on the Compression of Asymmetry of Sparsity” tackles the formidable challenge of managing the deluge of genomic data. It presents a novel compression algorithm, CA_SAGM, specifically designed for sparse asymmetric gene mutations (Ding et al.). This development is particularly pertinent for massive genomic databases like The Cancer Genome Atlas (TCGA), where efficient data handling is paramount. The study’s comparative analysis of CA_SAGM with other algorithms underscores the critical role of data compression in navigating the complexities of large-scale genomic datasets.

## Advancing genomic research through computational estimation techniques

Building on the theme of computational innovation, the paper “A Noise-tolerance Learning Method for Efficiently Estimating Open Chromatin Regions via cfDNA sequencing data” focuses on open chromatin regions (OCRs), crucial for understanding cellular functions and gene expression (Ren et al.). The introduction of OCRFinder, a learning-based, noise-tolerant approach, marks a significant stride in addressing the dynamic challenges of chromatin accessibility in cfDNA-seq data. By integrating ensemble learning and semi-supervised strategies, this study exemplifies the importance of sophisticated computational methods in genomic research, especially in areas like chromatin accessibility.

## Genomic data in clinical and prognostic applications

Shifting the spotlight to clinical implications, “Development and validation of focal adhesion-related genes signature in gastric cancer” illustrates the power of genomic data in disease prognosis (Zhao et al.). This paper offers a prognostic signature based on focal adhesion-related genes, showcasing how genomic data can be instrumental in identifying critical prognostic genes for gastric cancer. This research bridges computational methodologies and genomic insights, enhancing our understanding of cancer biology and providing valuable tools for cancer prognosis.

## Exploring disease mechanisms through gene modification analysis

The issue concludes with “Comprehensive Analysis of Key m5C Modification-Related Genes in Type 2 Diabetes,” which delves into the role of 5-methylcytosine (m5C) RNA methylation in type 2 diabetes (T2D) (Song et al.). Employing a variety of computational techniques, including LASSO regression and Gene Set Enrichment Analysis, the study sheds light on the molecular mechanisms of T2D. It highlights potential biomarkers and therapeutic targets, demonstrating the utility of genomic data in deciphering the complexities of disease processes.

Collectively, these articles represent the multifaceted applications of computational techniques in genomic data analysis. From optimizing data storage and processing to enhancing disease prognosis and understanding molecular mechanisms, these studies underscore the transformative impact of computational methods in the era of genomic big data. The innovations and insights showcased in this Research Topic are set to significantly shape future research and applications in genomics, bridging computational prowess with biological discovery.

## References

[B1] Ahmed I. Jeon G. Piccialli F. (2022). From artificial intelligence to explainable artificial intelligence in industry 4.0: a survey on what, how, and where. IEEE Trans. Industrial Inf. 18, 5031–5042. 10.1109/tii.2022.3146552

[B2] Caudai C. Galizia A. Geraci F. Le Pera L. Morea V. Salerno E. (2021). Ai applications in functional genomics. Comput. Struct. Biotechnol. J. 19, 5762–5790. 10.1016/j.csbj.2021.10.009 34765093 PMC8566780

[B3] Diaz-Flores E. Meyer T. Giorkallos A. (2022). “Evolution of artificial intelligence-powered technologies in biomedical research and healthcare,” in Smart biolabs of the future (Springer), 23–60. 10.1007/10_2021_189 35262750

[B4] Gheibi O. Weyns D. Quin F. (2021). Applying machine learning in self-adaptive systems: a systematic literature review. ACM Trans. Aut. Adapt. Syst. (TAAS) 15, 1–37. 10.1145/3469440

[B5] Hassija V. Chamola V. Mahapatra A. Singal A. Goel D. Huang K. (2023). Interpreting black-box models: a review on explainable artificial intelligence. Cogn. Comput., 1–30. 10.1007/s12559-023-10179-8

[B6] Jiao R. Nguyen B. H. Xue B. Zhang M. (2023). A survey on evolutionary multiobjective feature selection in classification: approaches, applications, and challenges. IEEE Trans. Evol. Comput., 1. 10.1109/tevc.2023.3292527

[B7] Liu Y. Wang Q. Xi J. (2022). Deepda-ace: a novel domain adaptation method for species-specific acetylation site prediction. Mathematics 10, 2364. 10.3390/math10142364

[B8] Mandal A. K. Sarma P. K. D. Dehuri S. (2023). A study of bio-inspired computing in bioinformatics: a state-of-the-art literature survey. Open Bioinforma. J. 16. 10.2174/18750362-v16-e230517-2022-17

[B9] Moczek A. P. Sears K. E. Stollewerk A. Wittkopp P. J. Diggle P. Dworkin I. (2015). The significance and scope of evolutionary developmental biology: a vision for the 21st century. Evol. Dev. 17, 198–219. 10.1111/ede.12125 25963198

[B10] Shi W. Chen W.-N. Kwong S. Zhang J. Wang H. Gu T. (2021). A coevolutionary estimation of distribution algorithm for group insurance portfolio. IEEE Trans. Syst. Man, Cybern. Syst. 52, 6714–6728. 10.1109/tsmc.2021.3096013

[B11] Shi W. Hu X.-M. Chen W.-N. (2022). An estimation of distribution algorithm with clustering for scenario-based robust financial optimization. Complex & Intelligent Syst. 8, 3989–4003. 10.1007/s40747-021-00640-2 PMC8897619 35284209

[B12] Ünal H. T. Başçiftçi F. (2022). Evolutionary design of neural network architectures: a review of three decades of research. Artif. Intell. Rev., 1–80.

[B13] Vandereyken K. Sifrim A. Thienpont B. Voet T. (2023). Methods and applications for single-cell and spatial multi-omics. Nat. Rev. Genet. 24, 494–515. 10.1038/s41576-023-00580-2 36864178 PMC9979144

[B14] Viriyasitavat W. Da Xu L. Dhiman G. Sapsomboon A. Pungpapong V. Bi Z. (2021). Service workflow: state-of-the-art and future trends. IEEE Trans. Serv. Comput. 16, 757–772. 10.1109/tsc.2021.3121394

[B15] Xi J. Deng Z. Liu Y. Wang Q. Shi W. (2023). Integrating multi-type aberrations from dna and rna through dynamic mapping gene space for subtype-specific breast cancer driver discovery. PeerJ 11, e14843. 10.7717/peerj.14843 36755866 PMC9901305

[B16] Xi J. Li A. Wang M. (2020a). HetRCNA: a novel method to identify recurrent copy number alternations from heterogeneous tumor samples based on matrix decomposition framework. IEEE/ACM Trans. Comput. Biol. Bioinforma. 17, 422–434. 10.1109/TCBB.2018.2846599 29994262

[B17] Xi J. Yuan X. Wang M. Li A. Li X. Huang Q. (2020b). Inferring subgroup-specific driver genes from heterogeneous cancer samples via subspace learning with subgroup indication. Bioinformatics 36, 1855–1863. 10.1093/bioinformatics/btz793 31626284

[B18] Yan J. Xi J. Yu Z. (2022). “A parametric model for clustering single-cell mutation data,” in 2022 IEEE international conference on bioinformatics and biomedicine (BIBM) (IEEE), 253–260.

[B19] Zhou T. Song Z. Sundmacher K. (2019). Big data creates new opportunities for materials research: a review on methods and applications of machine learning for materials design. Engineering 5, 1017–1026. 10.1016/j.eng.2019.02.011

